# A Unique Comprehensive Model to Screen Newborns for Severe Combined Immunodeficiency—An Ontario Single-Centre Experience Spanning 2013–2023

**DOI:** 10.3390/genes15070920

**Published:** 2024-07-15

**Authors:** Abdulrahman Al Ghamdi, Jessica Willett Pachul, Azhar Al Shaqaq, Meghan Fraser, Abby Watts-Dickens, Nicole Yang, Linda Vong, Vy H. D. Kim, Victoria Mok Siu, Anne Pham-Huy, Rae Brager, Brenda Reid, Chaim M. Roifman

**Affiliations:** 1 Division of Immunology & Allergy, Department of Pediatrics, Hospital for Sick Children, University of Toronto, Toronto, ON M5S 1A1, Canada; 2 Department of Pediatrics, King Faisal Specialist Hospital and Research Center Ar Rawdah, Jeddah 23433, Saudi Arabia; 3 Newborn Screening Program, Department of Clinical and Metabolic Genetics, Hospital for Sick Children, University of Toronto, Toronto, ON M5S 1A1, Canada; 4 Department of Molecular Genetics, University of Toronto, Toronto, ON M5S 1A1, Canada; 5 Canadian Centre for Primary Immunodeficiency, Hospital for Sick Children, University of Toronto, Toronto, ON M5S 1A1, Canada; 6 Division of Medical Genetics, Department of Pediatrics, London Health Sciences Centre, Western University, London, ON N6A 5A5, Canada; 7 Division of Infectious Diseases, Immunology and Allergy, Children’s Hospital of Eastern Ontario, Faculty of Medicine, University of Ottawa, Ottawa, ON K1N 6N5, Canada; 8 Division of Rheumatology, Immunology, and Allergy, Department of Paediatrics, McMaster Children’s Hospital, McMaster University, Hamilton, ON L8S 4L8, Canada

**Keywords:** newborn screening, severe combined immunodeficiency, SCID, leaky SCID, T cell deficiency, inborn errors of immunity, T cell receptor excision circles, TRECs, primary immunodeficiency, PID

## Abstract

Background: Severe combined immunodeficiency (SCID) is a life-threatening genetic disorder caused by critical defects of the immune system. Almost all cases are lethal if not treated within the first two years of life. Early diagnosis and intervention are thus essential for improving patient outcomes. In 2013, Ontario became the first Canadian province to perform newborn screening (NBS) for SCID by T cell receptor excision circles (TRECs) analysis, a surrogate marker of thymic function and lymphocyte maturation. Methods: This retrospective study reports on nearly 10 years of NBS for SCID at a quaternary referral centre. Results: From August 2013 to April 2023, our centre’s densely populated catchment area flagged 162 newborns with low TRECs levels, including 10 cases with SCID. Follow-up revealed other causes of low TRECs, including non-SCID T cell lymphopenia (secondary/reversible or idiopathic causes, and syndromic conditions) and prematurity. A small number of cases with normal repeat TRECs levels and/or T cell subsets were also flagged. Province-wide data from around this period revealed at least 24 diagnosed cases of SCID or Leaky SCID. Conclusions: This is the first report of NBS outcomes in a Canadian province describing the causative genetic defects, and the non-SCID causes of a positive NBS for SCID.

## 1. Introduction

Severe combined immunodeficiency (SCID) is a spectrum of inborn errors of immunity (IEI) characterized by profound T cell deficiency resulting in loss of adaptive immunity. Left untreated, infants with SCID develop chronic diarrhea, fail to thrive, and die due to overwhelming bacterial, fungal, or viral infections within the first 2 years of life [1]. T cells are essential for coordinating the cellular and humoral arms of the immune system and defects in their early development can result in dysregulation of other immune cell types, including B cells and NK cells [1]. Of the 485 genes associated with IEI (which include combined immunodeficiencies and conditions associated with antibody defects, immune dysregulation, phagocyte dysfunction, intrinsic and innate immunity, autoinflammatory disorders, complement deficiencies, bone marrow failure, and phenocopies of IEI), defects in 19 genes are known to cause SCID [2]. Notably, these genes are critically involved in T cell development and signal transduction (*IL2RG*, *JAK3*, *IL7R*, *PTPRC*, *CD3C*, *CD3E*, *CD3Z*, *LAT*, *LCP2*, *RAC2*), DNA cleavage/repair mechanisms required for generation of diverse T cell receptor repertoires (*RAG1*, *RAG2*, *DCLRE1C*, *PRKDC*, *NHEJ1*, *LIG4*), as well as metabolic defects (*ADA*, *AK2*). In some cases, ‘leaky SCID’ (hypomorphic SCID) due to defects in the above genes can cause SCID-like symptoms but with measurable (albeit low) T cell counts, whereas an autosomal recessive form of SCID, Omenn Syndrome, is characterised by autoimmune and allergic inflammation with severe erythroderma, enlarged lymphoid tissue, increased IgE, and eosinophilia. In many cases, the only curative treatment for SCID is haematopoietic stem cell transplantation (HSCT), in which dysfunctional haematopoietic stem cells are replaced with healthy donor cells, allowing for the reconstitution of a functional immune system. Gene therapy or enzyme replacement therapy may also be considered for certain conditions [3].

The inclusion criteria for newborn screening (NBS) requires the availability of (i) a reliable, low-cost yet high throughput test, (ii) definitive follow-up investigations that enable identification of true positives from false positives, and (iii) effective treatment that if administered during the presymptomatic period leads to significantly improve outcomes. Indeed, SCID satisfies the above given that most patients with SCID are asymptomatic at birth (due to the presence of maternally transferred antibodies), and early neonatal diagnosis and curative treatment improve survival and outcome [4,5]. The NBS test for SCID relies on the measurement of T cell receptor excision circles (TRECs) in dried blood spots collected from newborns [6]. TRECs are DNA byproducts of T cell receptor production in the thymus and are a marker of the recent development of mature T cells. High levels of TRECs are seen in healthy newborns, as they make a diverse population of naïve T cells with different T cell receptors, whereas they are low or absent in patients with SCID [6]. Although quite sensitive in detecting SCID, a low TREC level is not specific, as other causes for lymphopenia such as prematurity, exposure to maternal immunosuppression, and non-severe combined immunodeficiencies may also lead to a positive screen [7].

NBS for SCID was first implemented as a pilot project in 2008 and 2009 in Wisconsin, Massachusetts, and the Navajo Reservation in the United States (US) [8,9,10]. The success of these pilot projects led to the Advisory Committee on Heritable Disorders in Newborns and Children recommending that screening for SCID be added to the Recommended Uniform Screening Panel (RUSP) of newborn screened diseases in the US. Since then, all US states, most Canadian provinces, and many other countries have added SCID to their NBS programs.

NBS for SCID in Ontario, Canada, was implemented in August 2013 [11]. The algorithm for follow-up evaluation of positive SCID NBS cases at the Hospital for Sick Children was published in 2017 [12]. Although some changes have since been made, such as in-house 22q11.2 deletion testing and the development of a separate algorithm for outpatient and inpatient newborns with positive SCID NBS, the overall workup and testing remain unchanged [13]. During this period, the TRECs cut-off level was also refined, initially from 0 copies/3 µL, and then increased to 25 copies/3 µL and later 75 copies/3 µL. At present, all Canadian provinces except for Newfoundland and Labrador have implemented NBS for SCID. Here, we report the outcomes of infants screened and/or referred to the Hospital for Sick Children, Toronto, Ontario, over the past 10 years.

## 2. Methods

### 2.1. Data Collection

This study was conducted according to the guidelines of the Declaration of Helsinki and approved by the Research Ethics Board (REB) of The Hospital for Sick Children (Protocol no. 1000003390, 1000011085, and 1000081700). Patient consent was waived for subjects who initially had a positive SCID NBS but were later discharged without a diagnosis of primary immunodeficiency, and some patients who underwent transplantation, in accordance with REB guidelines. Informed consent was obtained from all other subjects involved in the study.

### 2.2. Newborn Screening

The NBS program in Ontario assesses for TRECs level, IKBKB (c.1292dupG), and Zap70 (c.1624-11G>A) founder mutations, as well as purine profiles (deoxyadenosine, deoxyguanosine, and guanosine). If the initial TRECs level, measured from a dried bloodspot taken at 24–48 h of life, falls below the cut-off (currently 75 copies/3 µL), the infant is referred to one of five retrieval centres in Ontario. A repeat TRECs determination is subsequently performed to verify the initial low TREC result.

**Figure 1:**
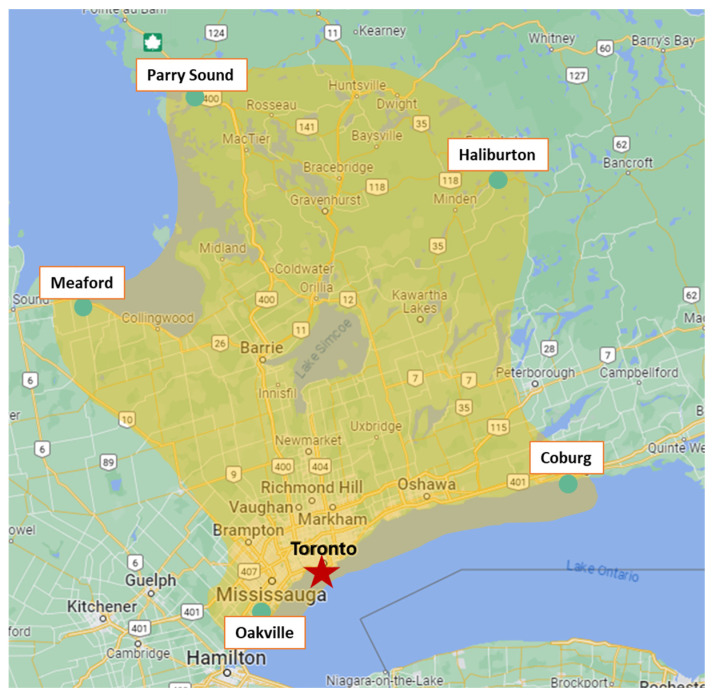
The Hospital for Sick Children’s catchment area for SCID newborn screening.

The Hospital for Sick Children, Toronto, Ontario, is the initial retrieval centre for infants with positive NBS and has a large and densely populated catchment area, as shown in Figure 1. A chart review was performed on all positive SCID NBS cases from its inception in August 2013 to the end of April 2023.

Our centre is also a quaternary referral centre for further management of most Ontario-based patients diagnosed with SCID, Leaky SCID, or Omenn Syndrome [14,15]. Some patients reside outside of our NBS catchment area and had their initial SCID NBS retrieval and follow-up at another location. Data regarding their SCID NBS results, affected genes, and diagnosis were collected by chart review between the period of August 2013 to the end of 2022.

### 2.3. Measurement of TREC levels

Determination of TREC levels from neonatal dried blood spots (Guthrie cards) was performed as previously described [16]. Briefly, neonatal blood was collected on Whatman 903 protein saver cards and sent to Newborn Screening Ontario (NSO), Ottawa, for analysis. A 3.2 mm spot was punched from the dried blood spots, placed into a 96-well U-bottom microtiter plate, and washed (×3) with 20 mM Tris-HCl (pH 9.0, ±0.5% Triton-X) at 37 °C on a microplate shaker. DNA was subsequently eluted in 50 µL Tris-HCl pH 9.0, 50 ng/L yeast t-RNA at 90 °C (40 min), and stored at −20 °C. Eight-point calibration curves were prepared using a serially diluted mini-gene (IDT) based on the TREC insert sequence [17] in washed packed red blood cells. For qPCR quantification of TREC levels, a reaction mix containing 0.9 µmol/L each of forward (TGCTGACACCTCTGGTTTTTGTAA) and reverse primers (GCCAGCTGCAGGGTTTAGG) that flank the δRec-ψJa TREC-specific splice junction, 0.25 umol/L TREC-specific hydrolysis probe (Quasar670-ATGCATAGGCACCTGCAC-BHQ2Plus), 5 µL DurAmp Master Mix, and 4.8 µL of DNA eluate was prepared. Template amplification conditions on an Applied Biosystems ViiA 7 Real-Time PCR System (384-well plate) are as follows: denaturation for 1 cycle at 95 °C (45 s) followed by 45 cycles of 95 °C (30 s) and 60 °C (1 min 30 s). Absolute quantification was performed using the Applied Biosystems QuantStudio v1.2 Sequence Detection System software. The acceptable ranges set for the slope were −3.32 (±2 Std Dev), Y-intercept Cq value of 41 (±2 Std Dev), and R^2^ value of 0.90 (±2 Std Dev).

### 2.4. SCID Diagnosis

Diagnosis of SCID, Leaky SCID, and Omenn Syndrome was made based on the PIDTC 2022 definition [18]. For other definitions, we conformed with the recommendations made by Blom et al. after a systematic review of NBS programs [19]. Confirmatory genetic testing/genetic diagnosis relied on sequencing of a panel of SCID/IEI genes (Blueprint Genetics Comprehensive Immune and Cytopenia Panel (642 genes); Prevention Genetics IEI/Primary Immunodeficiency Panel (609 genes); and NSO SCID/Primary Immune Deficiency Panel (251 genes)).

## 3. Results

### 3.1. SCID NBS within Catchment Area

**Figure 2:**
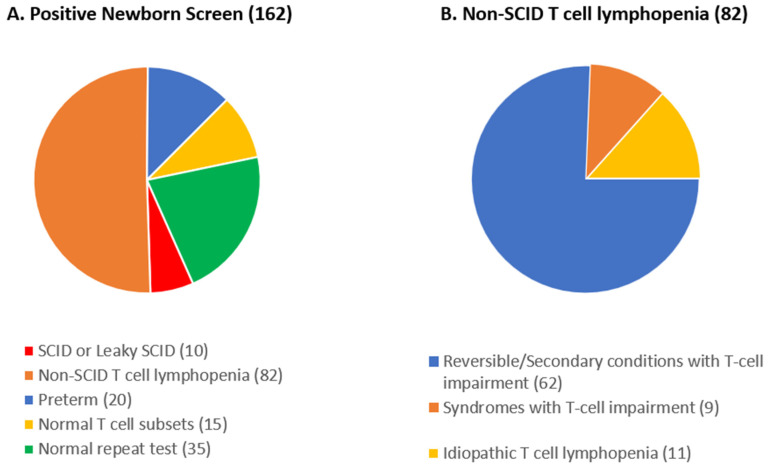
Breakdown of positive newborn screening cases in the Hospital for Sick Children catchment area by cause.

The total number of positive NBS cases (TREC < 75 copies/3 µL) for SCID in our catchment area from August 2013 up to April 2023 was 162. Of this group, 10 patients (6.2%) were eventually diagnosed with SCID or Leaky SCID (designated as patients 1–10, Table 1). A breakdown of other causes of positive SCID NBS results is shown in Figure 2A.

In total, 82 patients, or 50.6%, had non-SCID T cell lymphopenia (Figure 2B). A reversible or secondary cause was found in 62 cases (75.6%). Perinatal stressors were the cause in 50 newborns. In 7 cases, exposure to maternal immunosuppression was the cause of the low TRECs, and the remaining 5 newborns underwent thymectomy as part of cardiac surgery prior to SCID NBS testing. Idiopathic T cell lymphopenia occurred in 11 patients, and 9 patients had other combined immunodeficiencies (reported as syndromes with T cell impairment). Five patients were diagnosed with DiGeorge syndrome, 2 with Ataxia Telangiectasia, 1 with FOXN1 deficiency, and 1 with Coronin 1A deficiency.

A total of 35 patients had normal repeat TRECs on a separate sample (i.e., false positive) and 15 had normal T cell subsets on further workup and were discharged in good condition.

### 3.2. SCID Infants Detected by NBS in Ontario

The total number of infants screened in the province of Ontario between August 2013 and the end of December 2022 was 1,348,152. During this time, 24 infants (including the 10 within our catchment area) flagged by a positive SCID NBS were eventually diagnosed with SCID or Leaky SCID and referred to our centre for management. In total, 14 infants fulfilled the criteria for typical SCID, and 10 fulfilled the criteria for Leaky SCID. None of the patients were diagnosed with Omenn syndrome; however, patients 2, 3, and 20 had Omenn syndrome features and were clinically treated as such, but did not fulfill the PIDTC 2022 diagnosis criteria. Only 4 of the 24 patients were female. No patients who were diagnosed with SCID had a normal NBS result. Of note, some infants may have been referred to other centres outside of Ontario for management (personal communications), and thus were not included in our study.

Table 1 shows the patients’ initial TREC levels, diagnoses, and affected genes. Sixpatients had ADA deficiency, 3 had IL2RG deficiency, 3 had CD3 Delta deficiency, 2 had Artemis deficiency (affecting *DCLRE1C*), and 1 each had variants impacting *NHEJ1*, *TRAC*, *RAC2*, and *RMRP*. Six patients do not have a known genetic diagnosis.

## 4. Discussion

Since the implementation of NBS for SCID in Ontario, the Hospital for Sick Children has diagnosed and/or treated 24 patients with SCID who subsequently underwent HSCT or gene therapy. In one case, the affected individual passed away before curative treatment was administered. Of note, although our centre is the only quaternary centre for SCID management in Ontario, we cannot be certain that there have not been babies with SCID who died before referral or were referred to an out-of-province or out-of-country centre directly. However, even given this, the rate of SCID or Leaky SCID based on the number of infants screened would be 1 in 56,173 screened newborns (1.78 per 100,000 newborns). This is in keeping with estimates determined prior to the introduction of SCID NBS, especially when accounting for Canadian First Nations, Métis, and Inuit children [20], and is similar to data from US-based SCID NBS programs [21].

A positive NBS SCID result can be distressing for parents/guardians and is a period marked by uncertainty while follow-up testing is performed. Evaluations include complete blood counts with differential, enumeration of lymphocytes, assessment of immunophenotyping and lymphocyte function, as well as screening for cytomegalovirus (CMV) infection. Mothers are advised to halt breastfeeding until their CMV status has been established and family members are advised to reduce the risk of exposure to infections by limiting the number of visitors, avoiding public places, and practicing good hand hygiene. Interaction of the infant with other young children should be avoided, and those in close contact with the infant should not receive live or live-attenuated vaccines. Depending on the outcome of the tests, infants may remain at home during the evaluation, or if results are consistent with SCID, admission under protective isolation may be recommended. Psychosocial support may be helpful in assisting families throughout this period [22].

In our study, all patients with SCID or Leaky SCID had TREC levels far below the cut-off (75 copies/3 µL) and most had undetectable TRECs (Table 1). The strict definition of Omenn syndrome in the most recent criteria, especially the need for a known pathogenic variant in genes associated with Omenn syndrome, meant that none of our patients were formally diagnosed with Omenn syndrome, despite three patients being treated as such. This finding may not be specific to our population. In a study to implement the 2022 PIDTC criteria on a known cohort of SCID patients, Dvorak, et al. found that 17 of 32 patients that had features of Omenn syndrome did not fulfill the criteria, although 7 of these patients had evidence of maternal engraftment [23].

X-linked SCID is known to be the most common type of SCID in North America, although screening programs have shown that it makes up a smaller proportion than pre-NBS estimates [21]. In our cohort, ADA deficiency was the most common cause of SCID or Leaky SCID, which was also reported in the Canadian national surveillance study performed prior to the implementation of SCID NBS [20]. This may be due to certain communities with high rates of consanguinity and/or founder mutations, including the Mennonite/Low-German-speaking population. Other genetic causes of SCID were evenly distributed and are most likely due to the small number of cases and the genetic diversity in our population.

IEIs other than SCID are known to be a more common cause of low TREC levels in newborns compared to SCID or Leaky SCID [24,25]. In the cohort from our catchment area, 20 patients (12.3%) who had a positive NBS were diagnosed with combined immunodeficiencies other than SCID, with the largest group having idiopathic T cell lymphopenia and no known genetic diagnosis. These patients are an important group for further research, as they may help us in understanding immune development or the discovery of new IEIs. Clinical follow-up and monitoring of these patients is important to ensure there is no progression of their immunodeficiency or signs of opportunistic infections.

Comparing outcomes with other screening programs is somewhat complicated, given the heterogeneity of definitions and data reported. For this reason, in 2022, Blom et al. recommended standardised terminology and uniform definitions for reporting SCID newborn screen results [19]. These recommendations suggested against the use of the term “false positive” and instead using more descriptive terms to report newborns with abnormal TRECs who do not have SCID. While most reports of newborn screening for SCID were from before these recommendations, recent reports from Germany and New Zealand have used these uniform terms [26,27].

The German program reported 1443 abnormal values, of which they identified 35 patients with SCID, Leaky SCID, or Omenn Syndrome, which is a rate of 2.4% of abnormal values. Similarly, the New Zealand program reported 101 abnormal values and 2 SCID patients, a rate of 1.98%. In our catchment area, the SCID to abnormal value rate was 10 to 162 or 6.1%; this significantly higher rate is likely, at least in part, due to our program excluding infants below 34 weeks gestational age from screening, whereas the other two programs do not, leading to many abnormal values caused by prematurity.

For non-SCID causes of abnormal values, the German program had 1022 newborns with normal repeat TRECs, of whom 589 were premature (<32 weeks) and 389 were admitted to the NICU. Our cohort only had 35 of 162 with normal repeat values. This again emphasises the effect of prematurity on TREC levels. The German program reported two newborns with reversible causes of severe T cell lymphopenia, whereas the New Zealand program reported seven with reversible T cell lymphopenia. Our cohort had a much higher rate of reversible or secondary causes of T cell lymphopenia at 62. This may be due to different cut-offs used for reporting this outcome, as we did not limit this outcome to only those with severe T cell lymphopenia and included all those below the cut-off value for age. As for syndromic causes of abnormal results, both programs reported DiGeorge as the most common syndrome, as was the case in our cohort.

The economic feasibility of NBS programs depends on many variables, including the cost of the screening program, the population being screened, and the presumed benefits of screening. Cost-effectiveness and cost–benefit analyses conducted in the USA, New Zealand, France, the UK, the Netherlands, and Australia have shown favorable results [28,29,30,31,32,33,34]. This is due to SCID NBS using already implemented dried blood spot screening programs for newborns, which decreased the cost significantly, as well as the likely prevention of complications and improved survival associated with early detection of SCID. While no similar analysis has been carried out in Canada, given the benefit shown in many similar populations, it is reasonable to assume that it is cost-effective in our population.

In summary, this study is the first to report Ontario outcomes of NBS for SCID and, overall, it reveals similar results to other SCID NBS programs. In addition, it highlights some distinct features of our population, including the estimated prevalence of SCID, a higher rate of ADA deficiency, and a higher incidence of idiopathic T cell lymphopenia. Reports of screening results from other Canadian provinces are needed to compare with ours, and further studies on the outcomes of these patients and how they relate to the pre-NBS era are needed.

## Figures and Tables

**Table 1:** Patients with SCID or Leaky SCID in Ontario, 2013–2022.

Patient	Sex	Initial TREC Level	PIDTC Diagnosis	Gene Affected
1	Male	0	SCID	*IL2RG*
2	Male	0	Leaky SCID	Unknown
3	Male	45	Leaky SCID	Unknown
4	Male	1	Leaky SCID	Unknown
5	Female	0	Leaky SCID	*NHEJ1*
6	Male	0	SCID	*DCLRE1C*
7	Male	0	Leaky SCID	*TRAC*
8	Male	0	SCID	Unknown
9	Male	8	SCID	*ADA*
10	Male	0	SCID	*RAC2*
11	Male	0	Leaky SCID	*ADA*
12	Male	31	Leaky SCID	*ADA*
13	Male	0	SCID *	*ADA*
14	Female	25	SCID	*ADA*
15	Male	0	SCID **	*IL2RG*
16	Male	0	SCID	*ADA*
17	Male	5	Leaky SCID	*RMRP*
18	Male	0	SCID	*CD3D*
19	Male	0	SCID ***	*IL2RG*
20	Female	0	Leaky SCID	Unknown
21	Female	0	SCID	*DCLRE1C*
22	Male	0	SCID	*CD3D*
23	Male	0	SCID	*CD3D*
24	Male	0	Leaky SCID	Unknown

*ADA*: Adenosine Deaminase, *CD3D*: CD3 Delta, *DCLRE1C*: DNA Cross-link Repair 1C, *IL2RG*: Interleukin 2 Receptor Subunit Gamma, *NHEJ1*: Non-homologous End Joining Factor 1, *RAC2*: Rac Family Small GTPase 2, *TRAC*: T Cell Receptor Alpha Constant, TREC: T cell excision circles. * Primary data not found; diagnosis made based on physician notes. ** Passed away prior to detailed workup, found to have IL2RG pathogenic variant post-mortem. *** Diagnosed prenatally due to family history.

## Data Availability

The data presented in this study are available on reasonable request from the corresponding author.
